# Artificial Intelligence Reading of Postoperative Cochlear Implant X-Rays

**DOI:** 10.3390/jcm15156108

**Published:** 2026-08-06

**Authors:** Mariam Aljehani, Faris Albassam, Ebtesam Aljohani, Fida AlMuhawas, Farid Alzhrani, Abdulrahman Hagr

**Affiliations:** 1 King Abdullah Ear Specialist Center (KAESC), King Saud University Medical City, King Saud University, Riyadh 12629, Saudi Arabia; 2 College of Medicine, King Saud University, Riyadh 12372, Saudi Arabia; 3 College of Computer Science and Engineering, Taibah University, Madinah 42353, Saudi Arabia

**Keywords:** cochlear implant, postoperative radiography, deep learning, convolutional neural network, electrode malposition, transfer learning, explainable artificial intelligence, Grad-CAM, quality assessment, otology, green cochlea, sustainability

## Abstract

**Background/Objectives**: Postoperative X-ray is the standard method for confirming cochlear implant (CI) electrode position, but interpretation varies between readers and subtle abnormalities can be missed. We evaluated a proof-of-concept deep learning framework for automated classification of CI electrode position on postoperative X-rays across four CNN architectures, each trained with and without class weighting, as an initial step toward determining whether such models warrant further clinical development. **Methods**: We analyzed 1033 radiographic regions of interest from 673 patients at a tertiary referral center. ResNet18, EfficientNet-B0, DenseNet121, and EfficientNet-B3 were evaluated using patient-level data splitting, transfer learning, and consensus ground truth from two independent specialists, assessed on an independent test set and through five-fold patient-level cross-validation, with Grad-CAM used to evaluate model attention. **Results**: DenseNet121 (non-weighted) achieved the highest AUC (0.932; 95% CI 0.836–1.000) and specificity of 98.6% (95% CI 95.3–99.6%), while EfficientNet-B3 (weighted) reached the highest sensitivity (69.2%; 95% CI 42.4–87.3%). Under cross-validation, ResNet18 (weighted) was the most consistent model (AUC 0.861 ± 0.030); single-split AUC values were optimistic by a mean of 0.069 across models. Grad-CAM confirmed electrode-focused attention in ResNet18 and DenseNet121. **Conclusions**: Multi-architecture CNNs showed potential to classify CI electrode position with high specificity and clinically interpretable attention, supporting further investigation as screening-support tools rather than autonomous diagnostic systems.

## 1. Introduction

Cochlear implantation (CI) is the standard surgical treatment for severe-to-profound sensorineural hearing loss, restoring auditory function through direct electrical stimulation of the cochlear nerve [1]. Electrode position within the cochlea directly affects hearing outcomes [2,3], and postoperative X-ray is routinely performed to confirm full insertion and identify abnormalities—tip fold-over, kinking, or extracochlear malposition—before the patient leaves the operating room [1,2,4,5].

Postoperative radiographs, however, are not straightforward to interpret. Subtle malposition patterns require subspecialist experience, and general radiology reports can miss them [1,2]. Narrative reporting also lacks the consistency needed for large-scale auditing or electrode-guided programming [1,2].

Deep learning, specifically convolutional neural networks (CNNs), has shown strong diagnostic performance across medical imaging tasks [6,7,8,9,10,11]. Gradient-weighted Class Activation Mapping (Grad-CAM) addresses the black-box concern in clinical AI by showing which image regions drive each prediction [12,13].

Preoperative planning has become an increasingly important complement to postoperative radiographic assessment. Otological planning software such as OTOPLAN (CASCINATION AG and MED-EL, Innsbruck, Austria) allows surgeons to measure cochlear duct length from preoperative computed tomography and select an electrode array matched to individual cochlear size [14]. This individualized approach is supported by the development of electrode arrays with variable insertion depths, such as the FLEX family (including FLEX24, FLEX28, and FLEX34), which were designed to minimize insertion trauma while allowing array length to be tailored to each patient’s cochlear anatomy [15]. Beyond surgical planning, the same anatomical data underpin anatomy-based fitting (ABF), a programming strategy that aligns the frequency allocation of each electrode with its true tonotopic location in the cochlea, in contrast to default frequency tables that assume a standard cochlear size [16]. ABF can be derived either preoperatively from CT-based OTOPLAN reconstructions (preopABF) or postoperatively from plain X-ray-based reconstructions once the array is in place (postopABF), with recent work demonstrating the feasibility and validity of X-ray-guided postoperative ABF as an alternative to CT-based planning [17,18]. Because postoperative X-rays already inform both malposition detection and, increasingly, ABF-based programming, an automated and reliable method for interpreting these images has relevance beyond quality assurance alone.

In this preliminary exploratory study, we evaluated a proof-of-concept deep learning framework for automated classification of CI electrode position on postoperative X-rays, benchmarking four CNN architectures against expert-consensus ground truth to explore which, if any, might generalise reliably enough to eventually support routine postoperative quality assessment.

## 2. Materials and Methods

### 2.1. Study Design and Setting

We conducted this retrospective diagnostic accuracy and agreement study at the King Abdullah Ear Specialist Center (KAESC), a tertiary referral center in Riyadh, Saudi Arabia. Institutional Review Board (IRB) approval was obtained (KAESC IRB, approval number: R26-IRB-334; date: 13 May 2026), and the study adhered to the principles of the Declaration of Helsinki. Informed consent was waived owing to the retrospective design.

### 2.2. Patient Population and Dataset

We retrospectively included all patients who underwent cochlear implantation at KAESC. Inclusion criteria were the availability of early postoperative X-rays (standardized cochlear/Stenver’s view, performed within 4 weeks of surgery) and corresponding clinical radiology reports. The standardized cochlear/Stenver’s view is a posteroanterior oblique skull radiographic projection (head rotated approximately 45° and angled caudally) that images the petrous temporal bone and cochlea en face, allowing the intracochlear electrode array to be visualized along its full basal-to-apical course [19,20].

### 2.3. Labeling Strategy

**Figure 1:**
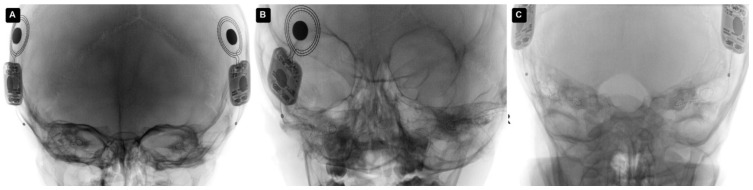
Representative bilateral postoperative cochlear implant radiographs: (**A**) partial insertion on the left side of the image and normal insertion on the right; (**B**) bilateral complete insertion; and (**C**) bilateral over-insertion.

We established a definitive reference standard (“ground truth”) through a masked review of a subset of 700 cases by two independent senior specialists, with discrepancies resolved by consensus discussion. Formal inter-rater agreement statistics were not computed; cases requiring consensus resolution were managed through joint specialist review until agreement was reached. This approach is consistent with established practice in diagnostic reference-standard development when a single ground truth is required from multiple reviewers. For analysis, the four-category label scheme was collapsed into a binary outcome, Normal (normal intracochlear full insertion) versus Abnormal (all other categories, including partial insertion, extracochlear malposition, and suspected abnormal trajectory), to maximize the sample available for model training. During the reference-standard process, primary labels included (1) normal intracochlear full insertion, (2) intracochlear partial insertion, (3) extracochlear/malposition, and (4) suspicion of abnormal trajectory. The four-category reference-standard labeling scheme and its corresponding binary grouping are summarized in Table 1. This was done because the individual abnormal subtypes were too infrequent to train reliable multiclass models; binary grouping maximised the abnormal sample available for learning and evaluation. We acknowledge that this collapse discards clinically meaningful distinctions—partial insertion and extracochlear malposition carry different surgical implications—and a multiclass formulation remains an objective for future work with a larger abnormal cohort. Representative postoperative radiographs showing the different electrode insertion positions are presented in Figure 1.

### 2.4. Image Preprocessing and Feature Extraction

**Figure 2:**
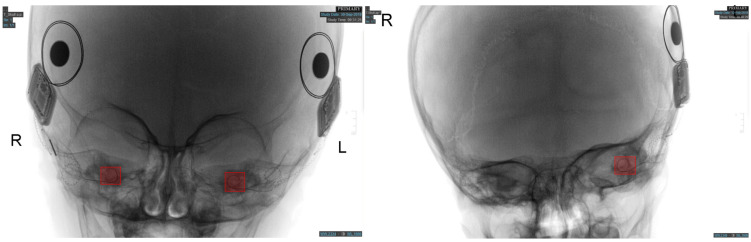
Representative early postoperative CI radiographs (standardized inverted view) with manually annotated ROI bounding boxes around the electrode array: bilateral (**left**) and unilateral (**right**) implantations. R and L denote right and left ears, respectively. Red boxes indicate the manually drawn regions of interest (ROI) encompassing the electrode array for each implant.

We manually annotated regions of interest (ROIs) corresponding to the cochlea and the electrode array using the LabelImg tool, and annotation data were exported as CSV files containing bounding-box coordinates and labels. The bounding-box coordinates (x, y, width, height) were then converted from percentage format to pixel values, based on the original image dimensions, using Python (version 3.10) within a Google Colab environment. Each ROI was cropped, resized to 256 × 256 pixels, and intensity-normalized before use as model input. Representative postoperative X-ray images with their corresponding annotated ROIs are presented in Figure 2.

ROI annotation was performed by an otology registrar (a supervised otolaryngology trainee); bounding boxes were placed based solely on the visible cochlear and electrode-array anatomy on each radiograph, without reference to the clinical radiology report or to the reference-standard normal/abnormal label, so the annotator was blinded to case status at the time of ROI placement.

### 2.5. Ai Model Development and Training

We evaluated four CNN architectures using transfer learning [21,22] within the PyTorch framework: ResNet18 [23], EfficientNet-B0 [24], DenseNet121 [25], and EfficientNet-B3 [24]. To address class imbalance (949 normal vs. 84 abnormal cases) [26], a non-weighted approach using standard cross-entropy loss was compared against a weighted strategy that applied a class-weighted loss function together with weighted random sampling to increase the representation of abnormal cases during training.

Dataset partitioning: We partitioned patients into training (70%), validation (15%), and independent testing (15%) sets using stratified sampling on patient-level labels. All ROIs belonging to the same patient, including bilateral images, were assigned to the same subset to prevent data leakage.

**Figure 3:**
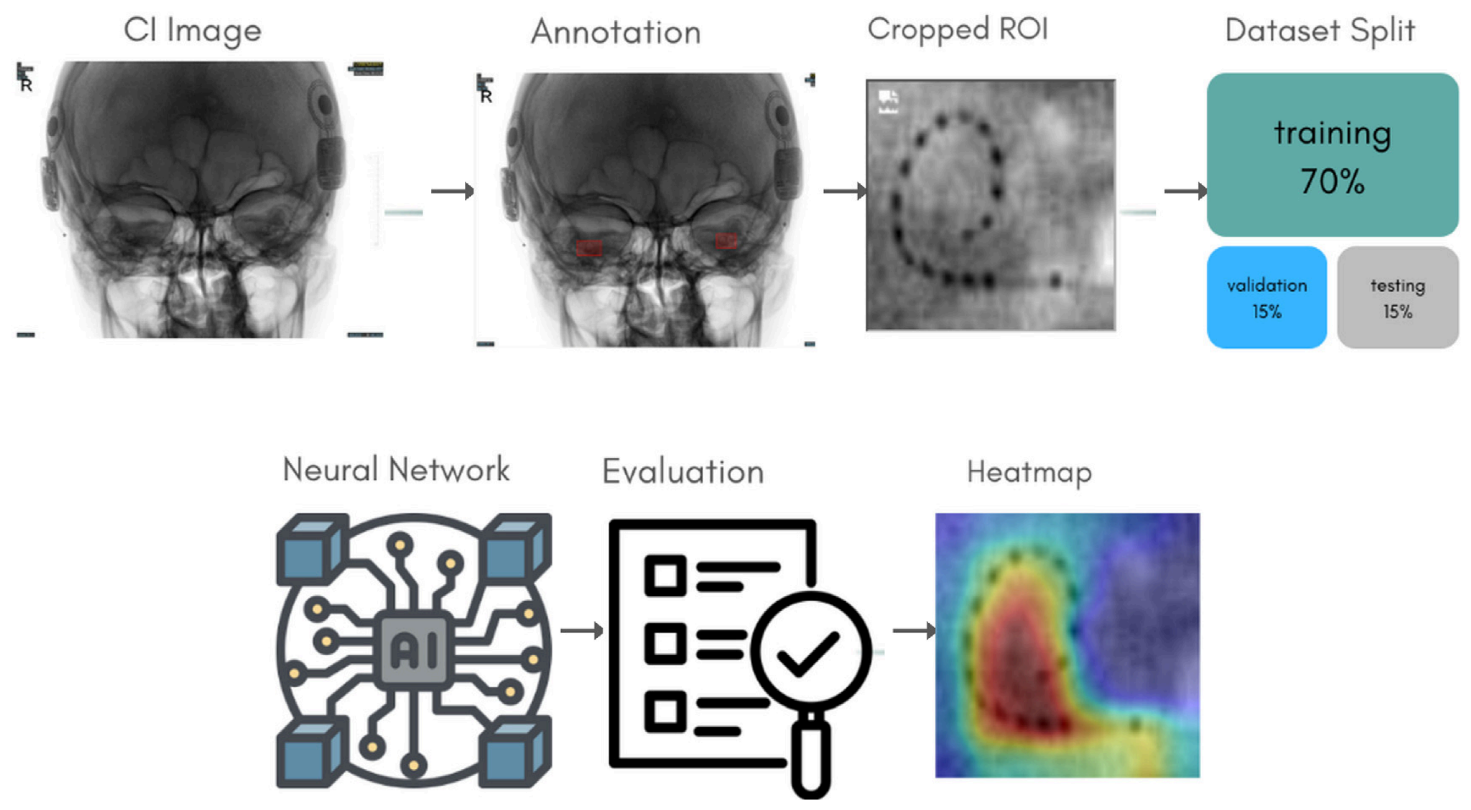
Overview of the model development pipeline, from ROI annotation and preprocessing through CNN training (with and without class weighting) to evaluation on the independent test set and five-fold cross-validation.

Training hyperparameters: We trained all models using the AdamW optimizer [27] with a learning rate of 1 × 10^−4^, a batch size of 16, an input size of 256 × 256 pixels, and 20 training epochs. The overall study workflow from image acquisition to model evaluation is summarized in Figure 3.

Classification threshold optimization: Thresholds were optimised on the validation set (range 0.30–0.50) by maximising the F1-score, balancing sensitivity and precision for abnormal-case detection.

### 2.6. Evaluation and Explainability

We evaluated model performance on the independent patient-level test set using accuracy, sensitivity, specificity, PPV, NPV, F1-score, and AUC; confusion matrices and ROC curves were generated for each configuration. Grad-CAM visualizations were used to assess whether each model attended to clinically relevant electrode regions.

## 3. Results

### 3.1. Primary Test Set Analysis

**Figure 4:**
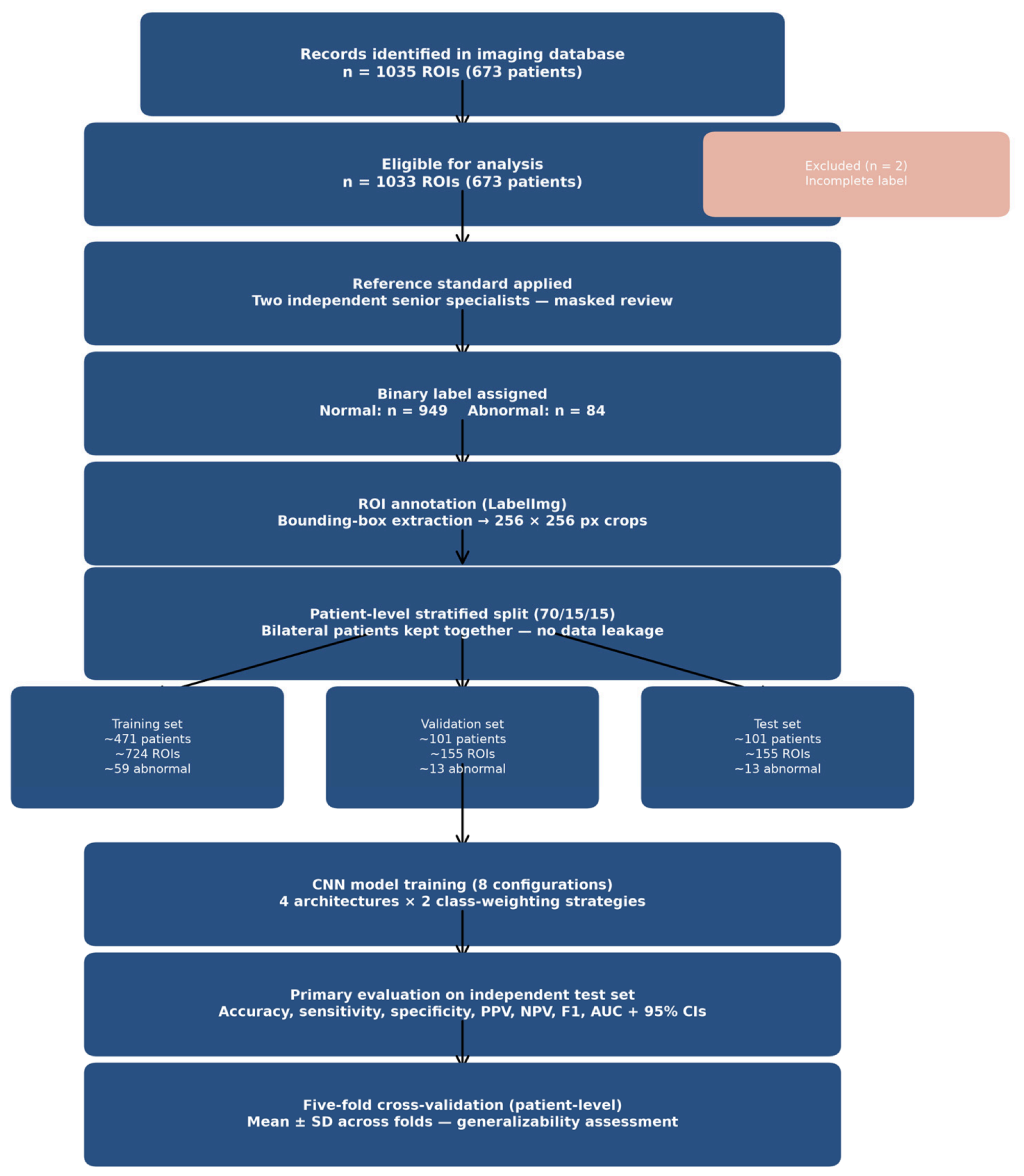
STARD-compliant patient flow diagram showing the study population from initial records through to statistical analysis. Approximate split counts are based on stratified patient-level partitioning (70/15/15).

A total of 673 of the 700 patients screened (1033 ROIs) met the inclusion criteria. Of these, 949 ROIs were labeled as normal electrode position and 84 as abnormal (prevalence 8.1%). Two cases with incomplete labels were excluded prior to analysis. After patient-level stratified partitioning, the training, validation, and test sets contained approximately 471, 101, and 101 patients, respectively (approximately 724, 155, and 155 ROIs; approximately 59, 13, and 13 abnormal cases per subset). The flow of patients from initial data collection through to final analysis is shown in Figure 4.

We evaluated eight model configurations on an independent patient-level test set, ResNet18, EfficientNet-B0, DenseNet121, and EfficientNet-B3, each trained with and without class weighting shown in Table 2.

DenseNet121 (non-weighted) performed best overall, with an accuracy of 94.3%, sensitivity of 46.2% (95% CI: 23.2–70.9%), specificity of 98.6% (95% CI: 95.3–99.6%), PPV of 75.0%, NPV of 95.4%, F1-score of 0.571, and the highest AUC of 0.932 (95% CI: 0.836–1.000). Full 95% CIs for all eight model configurations are presented in Table 2. Grad-CAM analysis showed attention localized to the mid-electrode only, supporting clinically relevant feature extraction.

ResNet18 (non-weighted) also demonstrated strong performance, with an accuracy of 93.7%, sensitivity of 46.2% (95% CI: 23.2–70.9%), specificity of 97.9% (95% CI: 94.3–99.3%), PPV of 66.7%, NPV of 95.3%, F1-score of 0.545, and an AUC of 0.818 (95% CI: 0.675–0.961). Its Grad-CAM activation was focused on the apical electrode only, again indicating clinically meaningful attention.

Among weighted models, EfficientNet-B3 (weighted) achieved the highest sensitivity (69.2%; 95% CI: 42.4–87.3%). It also showed an accuracy of 90.6%, specificity of 92.5% (95% CI: 87.4–95.9%), PPV of 45.0%, NPV of 97.1%, F1-score of 0.545, and a strong AUC of 0.919 (95% CI: 0.816–1.000), with Grad-CAM focusing on the apical electrode only, suggesting improved interpretability in the weighted setting.

DenseNet121 (weighted) showed improved sensitivity compared with its non-weighted counterpart, achieving 53.8% sensitivity (95% CI: 29.1–76.8%), with an accuracy of 90.6%, specificity of 93.8% (95% CI: 89.1–96.8%), PPV of 43.8%, NPV of 95.8%, F1-score of 0.483, and an AUC of 0.908 (95% CI: 0.798–1.000), and focal basal-electrode-only attention. For ResNet18 (weighted), sensitivity remained 46.2% (95% CI: 23.2–70.9%), with an accuracy of 86.2%, specificity of 89.7% (95% CI: 83.5–93.4%), PPV of 28.6%, NPV of 94.9%, F1-score of 0.353, and an AUC of 0.890 (95% CI: 0.772–1.000); weighting did not improve sensitivity and reduced precision and specificity, although Grad-CAM still showed predominantly electrode attention.

**Figure 5:**
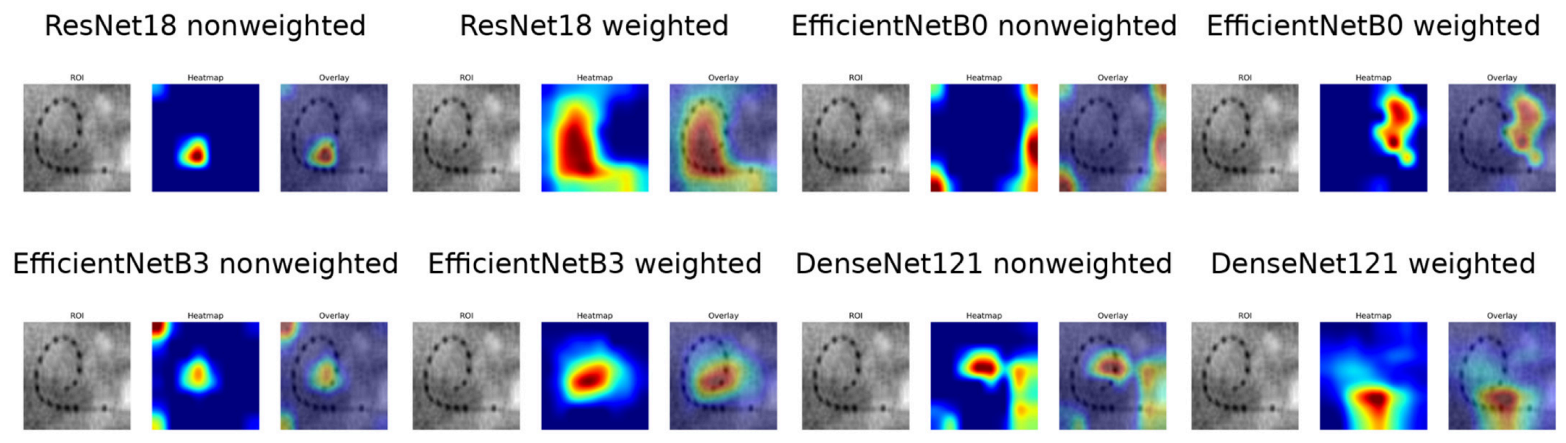
Grad-CAM visualizations for all eight model configurations (original image, heatmap, and overlay). ResNet18 and DenseNet121 consistently localize attention on the electrode array, whereas the EfficientNet-B0 and non-weighted EfficientNet-B3 models attend to peripheral, non-electrode regions.

The EfficientNet-B0 models showed high specificity but weaker sensitivity and less clinically meaningful attention patterns. EfficientNet-B0 (non-weighted) achieved an accuracy of 93.7%, specificity of 98.6% (95% CI: 95.3–99.6%), and PPV of 71.4% but a sensitivity of only 38.5% (95% CI: 17.7–64.5%) and an AUC of 0.780 (95% CI: 0.628–0.932), with peripheral non-electrode focus. EfficientNet-B0 (weighted) slightly improved sensitivity to 46.2% (95% CI: 23.2–70.9%) and increased the AUC to 0.902 (95% CI: 0.789–1.000), but interpretability remained suboptimal, with partial non-electrode attention. Similarly, EfficientNet-B3 (non-weighted) demonstrated the lowest sensitivity among all models (30.8%, 95% CI: 12.7–57.6%), despite good specificity (96.6%, 95% CI: 92.5–98.6%) and an AUC of 0.876 (95% CI: 0.752–1.000), with peripheral non-electrode attention suggesting reliance on less relevant image regions. Representative Grad-CAM heatmaps for all eight model configurations are presented in Figure 5.

**Figure 6:**
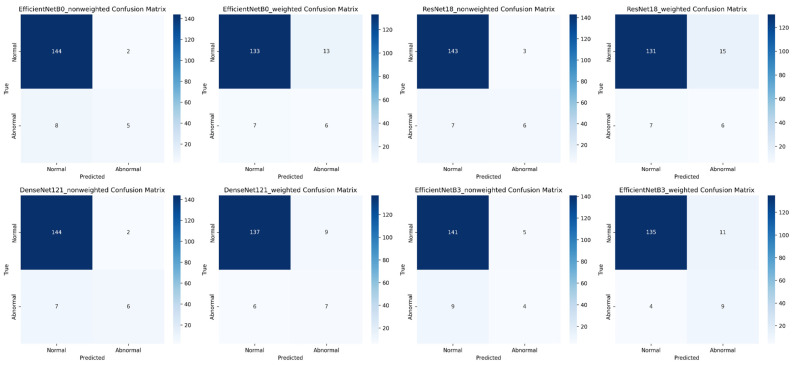
Confusion matrices for all eight model configurations on the independent patient-level test set.

**Figure 7:**
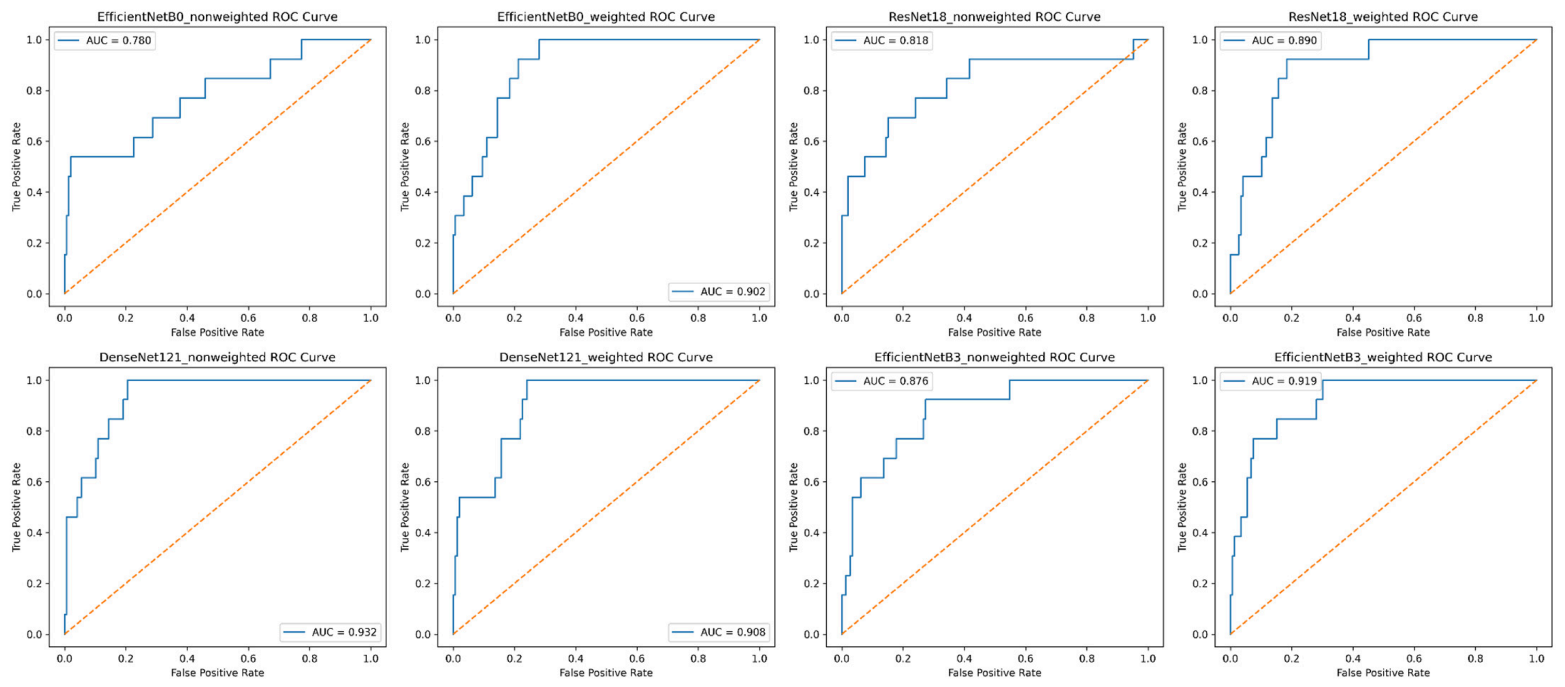
Receiver operating characteristic (ROC) curves and corresponding AUC values for all eight model configurations.

Optimal classification thresholds varied across models, ranging from 0.30 to 0.50. In general, lower thresholds in weighted models were associated with increased sensitivity, whereas the threshold of 0.50 in non-weighted models favored higher specificity and precision. Overall, DenseNet121 (non-weighted) provided the best balance of discrimination and interpretability, whereas EfficientNet-B3 (weighted) provided the highest abnormal-case sensitivity. Confusion matrices for all eight model configurations on the independent test set are presented in Figure 6, and the corresponding receiver operating characteristic curves are shown in Figure 7.

### 3.2. Five-Fold Cross-Validation

To assess generalizability, we performed five-fold stratified cross-validation at the patient level. All 673 patients were partitioned into five folds, with each fold serving once as the test set (~135 patients per fold, approximately 16–17 abnormal cases per fold). The same eight model configurations were retrained and evaluated in each fold, using identical hyperparameters, threshold-tuning strategy, and augmentation pipeline as in the primary analysis. Results are reported as mean ± standard deviation across folds shown in Table 3.

DenseNet121 (non-weighted) showed the most stable specificity across folds (97.3% ± 1.1%) and a mean AUC of 0.835 ± 0.061, the lowest variability among non-weighted models. Its mean sensitivity was 34.4% ± 15.1%, with a mean F1-score of 0.404 ± 0.142, confirming strong and stable precision in ruling out abnormal cases while maintaining clinically relevant discrimination.

ResNet18 (weighted) emerged as the most stable model overall, achieving an AUC of 0.861 ± 0.030, the lowest standard deviation across all eight configurations, with mean sensitivity of 57.7% ± 22.8% and specificity of 87.6% ± 6.6%. This consistency suggests that ResNet18 with class weighting generalizes more reliably across different patient subsets than more complex architectures.

Among all weighted models, DenseNet121 (weighted) achieved the highest mean sensitivity at 58.4% ± 27.1%, and EfficientNet-B3 (weighted) showed the second-highest AUC at 0.855 ± 0.117. However, both exhibited substantial fold-to-fold variability—standard deviations of 27.1% and 26.8% for sensitivity, respectively—reflecting instability attributable to the small number of abnormal cases per test fold (~16–17).

Cross-validation AUC was consistently lower than the single-split test-set AUC across all models (mean reduction: 0.069, range: 0.022–0.120). The largest discrepancy was observed for DenseNet121 (non-weighted), whose single-split AUC of 0.932 reduced to 0.835 ± 0.061 under cross-validation, and for EfficientNet-B0 (weighted), which dropped from 0.902 to 0.782 ± 0.110. In contrast, ResNet18 (weighted) remained most stable, declining only from 0.890 to 0.861 ± 0.030. These findings indicate that single-split evaluations on small imbalanced datasets are susceptible to optimistic bias and that cross-validation provides a more conservative and realistic estimate of generalization performance.

The wide sensitivity standard deviations observed across all models are consistent with the limited number of abnormal cases per fold (~16–17), which inevitably leads to high variability in true-positive counts. This confirms that the primary limitation of this dataset is not the model architecture but rather the size of the abnormal class, and prospective data collection targeting a larger abnormal cohort would substantially stabilize sensitivity estimates.

### 3.3. Statistical Model Comparisons

To formally compare model performance, we performed pairwise AUC comparisons using paired *t*-tests across the five cross-validation folds (a paired approach was valid because each model was evaluated on identical patient subsets per fold). A paired *t*-test on five fold-level AUC summary values is a repeated-measures comparison of fold-level summary estimates and is not equivalent to the DeLong test, which instead compares correlated AUCs computed from individual-patient predicted probabilities and directly models their covariance structure; we therefore report the fold-level paired *t*-test for what it is rather than as a DeLong-equivalent procedure. Individual-patient predicted probabilities from each fold were not retained for every model variant, precluding a retrospective DeLong analysis or a patient-level bootstrap on pooled out-of-fold predictions; this is noted as a limitation (Section 4). Because 8 model variants were compared across 28 pairwise AUC tests, we additionally applied a Holm–Bonferroni correction for multiple comparisons. For weighted versus non-weighted comparisons within each architecture, paired *t*-tests were also applied to sensitivity and specificity across folds.

Among all 28 pairwise AUC comparisons, one nominally reached statistical significance before correction: ResNet18 (weighted) demonstrated higher AUC than EfficientNet-B0 (non-weighted) (mean AUC 0.861 vs. 0.737; raw *p* = 0.016). However, after Holm–Bonferroni correction for the 28 comparisons performed, this difference did not remain significant (adjusted *p* = 0.43), and no pairwise AUC comparison survived multiplicity correction. This absence of statistically robust pairwise differences is expected given the low statistical power inherent to five-fold cross-validation on a dataset with a small abnormal cohort (~16–17 abnormal cases per test fold) and is consistent with the overlapping confidence intervals observed across models. We report this corrected result rather than the previously stated single nominally significant comparison, as the uncorrected comparison materially overstated the evidence for a performance difference between models.

For within-architecture weighted versus non-weighted comparisons (12 tests: 4 architectures × 3 metrics—AUC, sensitivity, specificity), EfficientNet-B0 showed a nominal improvement in sensitivity with class weighting (mean 49.8% vs. 29.0%; raw *p* = 0.016), but this did not remain significant after Holm–Bonferroni correction for the 12 comparisons (adjusted *p* = 0.20). No architecture demonstrated a statistically robust difference in AUC, sensitivity, or specificity between weighted and non-weighted variants after correction, indicating that any effect of class weighting observed at the uncorrected level should be interpreted as hypothesis-generating rather than confirmatory, consistent with the limited number of abnormal cases available per fold.

These statistical findings support a nuanced interpretation: rather than identifying a single definitively superior model, the results demonstrate that most models perform within a statistically comparable range under cross-validation (AUC 0.74–0.86), with ResNet18 (weighted) offering the most consistent generalization (lowest AUC standard deviation: 0.030) and DenseNet121 (non-weighted) offering the most stable high specificity (97.3% ± 1.1%). The choice between models should therefore be guided by the intended clinical application—prioritizing specificity for rule-out screening or sensitivity for abnormality detection—rather than by marginal numerical differences that do not reach statistical significance (Table 4).

## 4. Discussion

The present study demonstrates that CNN architecture and training strategy substantially influence automated CI electrode classification performance on postoperative X-rays. Although DenseNet121 achieved the highest single-split AUC, weighted ResNet18 showed the most consistent performance across cross-validation, highlighting the importance of evaluating model robustness rather than relying on a single test split.

Several groups have applied deep learning to cochlear implant imaging. Prior efforts have largely focused on CT-based three-dimensional electrode localization: Zhao et al. [6] proposed atlas-based electrode localization in postoperative CT, and Ma et al. [7] subsequently extended this to a unified deep learning framework achieving sub-millimetre localization accuracy across 561 clinical cases. The present work differs from these approaches in several important respects. First, it operates on conventional postoperative X-rays rather than CT, which are far more widely available and routinely performed at most cochlear implant centers without additional radiation exposure or cost. Second, the task focuses on binary quality classification rather than continuous electrode localization, addressing a complementary clinical need: rapid flagging of potential malpositions for specialist review. Third, the use of region-of-interest-based classification on grayscale images presents a distinct set of challenges—notably the absence of three-dimensional structural context—that the present multi-architecture comparison was designed to investigate. Together, these studies suggest that X-ray-based and CT-based AI approaches are not competing but complementary, with X-ray screening suitable for high-volume postoperative quality assurance and CT-based localization appropriate for detailed pre-programming assessment.

On the primary test set, DenseNet121 (non-weighted) achieved the highest AUC and F1-score, along with the best specificity and PPV. Grad-CAM activation was confined to the mid-electrode, confirming anatomically relevant feature use. Under five-fold cross-validation (Section 3.2), however, ResNet18 (weighted) was the most consistently generalizable model (CV AUC: 0.861 ± 0.030, the lowest standard deviation across all configurations), suggesting that the DenseNet121 single-split AUC of 0.932 may reflect a favorable test-set composition rather than a definitively superior architecture.

ResNet18 (non-weighted) matched DenseNet121 on sensitivity and showed similarly high specificity. Grad-CAM attention was confined to the apical electrode. A relatively simple architecture, trained with the right data, can still learn clinically meaningful features.

Class weighting improved sensitivity in some architectures but not others. EfficientNet-B3 (weighted) achieved the highest sensitivity overall, which matters most in a screening context where missing an abnormality is the primary concern. However, this gain came at the expense of lower PPV and reduced specificity, reflecting more false-positive predictions. A similar pattern was observed with DenseNet121 (weighted), where sensitivity improved but F1-score and PPV declined relative to the non-weighted model.

The likelihood ratios clarify how these models would behave at the point of care. Positive likelihood ratios were high for the non-weighted models—33.0 for DenseNet121, 27.5 for EfficientNet-B0, and 22.0 for ResNet18—meaning that a positive prediction substantially raises the probability of a true malposition and is well suited to flagging cases for specialist re-read. Negative likelihood ratios, by contrast, ranged from 0.33 to 0.72 and never approached the threshold (below 0.1) needed to confidently exclude an abnormality. In practical terms, the models are useful for ruling in suspected malpositions but cannot yet rule them out; a negative result should not be used to clear a case from further review. This positions the framework as a triage aid that raises specialist attention to higher-risk radiographs, rather than a replacement for expert interpretation.

Optimal thresholds varied from 0.30 to 0.50, reflecting differences in probability calibration. Weighted models with lower thresholds gained sensitivity; non-weighted models at 0.50 favored specificity. This finding supports the use of model-specific threshold tuning rather than a uniform cutoff.

The Grad-CAM results added important context beyond the numerical metrics. DenseNet121 and ResNet18 consistently attended to electrode regions, with activation localised to the apical, mid, or basal portion depending on the configuration. EfficientNet-B0 (non-weighted) and EfficientNet-B3 (non-weighted) frequently attended to peripheral, non-electrode regions, suggesting reliance on spurious image features. Although weighting improved interpretability in EfficientNet-B3, resulting in apical-electrode-only attention, EfficientNet-B0 remained less reliable even after weighting, showing only partial non-electrode attention. These findings emphasize that strong numerical performance alone is not sufficient for clinical AI models unless the underlying attention is anatomically meaningful.

Patient-level splitting prevented data leakage from bilateral cases. The validity of the reported diagnostic estimates was further supported by sample-size considerations grounded in established methodology for diagnostic test studies, namely Buderer and Flahault et al. [28,29], which account for disease prevalence and design accuracy when estimating sensitivity, specificity, and AUC. The inclusion of Grad-CAM analysis provided an important interpretability layer beyond conventional performance metrics.

Akinci D’Antonoli et al. [30], in a systematic review of large language model applications in radiology, highlight the emerging potential of such models to convert imaging findings into structured clinical reports. A natural extension of the present framework would integrate a downstream language model capable of translating binary electrode classification output into a structured narrative quality-assessment report, streamlining the postoperative documentation workflow.

The study has clear limitations. The dataset remained imbalanced, with relatively few abnormal cases (84 ROIs across 76 patients), which contributed to the moderate sensitivity seen in most models and the wide confidence intervals and cross-validation standard deviations on sensitivity estimates. Based on the Buderer formula [28], achieving a sensitivity estimate with a 95% confidence interval width of ±15 percentage points at an expected sensitivity of 70% and an abnormal prevalence of approximately 8% would require a minimum of approximately 200 abnormal cases in the test set—substantially more than was available in the present study. Future prospective data collection should prioritize abnormal-case accrual to achieve stable sensitivity estimates. Additionally, classification thresholds were optimized on the validation set to maximize the F1-score; while this approach is methodologically standard, optimal thresholds identified on held-out validation data may not generalize directly to prospective deployment settings, and threshold recalibration on local data would be advisable before clinical use. The analysis also relied on manually annotated regions of interest, which may limit scalability in routine clinical use. Additionally, ROI placement reproducibility (e.g., inter- or intra-annotator bounding-box overlap) was not formally assessed in this proof-of-concept study and should be quantified (for example, with intersection-over-union between repeated annotations) in future work. Because the present study was designed as an initial feasibility investigation of image-level classification rather than as a deployable end-to-end pipeline, we deliberately scoped the task to classification of an expert-defined ROI; automated, annotation-free localization of the cochlea and electrode array (e.g., via an object-detection or segmentation front end) is an explicit next step before the framework could function as a fully automated, end-to-end system operating on unprocessed radiographs. Finally, formal inter-rater agreement statistics (e.g., Cohen’s kappa) were not computed for the reference standard, as individual reviewer labels prior to consensus were not retained; future studies should record and report reviewer-level labels to allow formal quantification of annotation reliability. Finally, all data were collected at a single tertiary referral center, and performance on images acquired with different hardware, acquisition protocols, or patient populations remains unknown. External validation at additional centers is a necessary prerequisite before any clinical deployment of these models.

## 5. Conclusions

This preliminary exploratory study demonstrates the potential of deep learning for automated postoperative CI X-ray assessment. Across four CNN architectures evaluated with and without class weighting, DenseNet121 (non-weighted) achieved the highest single-split AUC and specificity, while weighted ResNet18 provided the most consistent performance under five-fold cross-validation, and EfficientNet-B3 (weighted) achieved the highest sensitivity. Grad-CAM visualizations confirmed that the best-performing models attended to anatomically relevant electrode regions rather than spurious image features, supporting the clinical plausibility of the classifications. These findings support the feasibility of AI-assisted screening for CI electrode position, with model choice guided by the intended clinical application (specificity for rule-out screening versus sensitivity for abnormality detection). However, the dataset’s limited number of abnormal cases, reliance on manual ROI annotation, and single-center design constrain generalizability. Larger multicentre datasets, prospective external validation, and formal inter-rater reliability assessment are essential before these models can be considered for clinical use.

## Figures and Tables

**Table 1:** Four-category reference-standard labeling scheme applied during masked expert review, and the binary grouping used for model training.

Reference-Standard Label	Binary Grouping (for Model Training)
Normal intracochlear full insertion	Normal
Intracochlear partial insertion	Abnormal
Extracochlear/malposition	Abnormal
Suspicion of abnormal trajectory	Abnormal

**Table 2:** Primary test-set performance metrics for all eight model configurations (four CNN architectures, each trained with and without class weighting). Weighted-model rows are marked with an asterisk (*).

Model	Threshold	Accuracy (%)	Sensitivity (%)	Specificity (%)	PPV (%)	NPV (%)	F1-Score	AUC	LR+	LR−	Grad-CAM Focus Area
ResNet18 (non-weighted)	0.50	93.7	46.2 [23.2–70.9]	97.9 [94.3–99.3]	66.7	95.3	0.545	0.818 [0.675–0.961]	22.0	0.55	Apical electrode only
ResNet18 (weighted) *	0.35	86.2	46.2 [23.2–70.9]	89.7 [83.5–93.4]	28.6	94.9	0.353	0.890 [0.772–1.000]	4.5	0.60	Predominantly electrode
EfficientNet-B0 (non-weighted)	0.50	93.7	38.5 [17.7–64.5]	98.6 [95.3–99.6]	71.4	94.7	0.500	0.780 [0.628–0.932]	27.5	0.62	Peripheral non-electrode focus
EfficientNet-B0 (weighted) *	0.45	87.4	46.2 [23.2–70.9]	91.1 [85.8–94.9]	31.6	95.0	0.375	0.902 [0.789–1.000]	5.2	0.59	Partial non-electrode attention
DenseNet121 (non-weighted)	0.50	94.3	46.2 [23.2–70.9]	98.6 [95.3–99.6]	75.0	95.4	0.571	0.932 [0.836–1.000]	33.0	0.55	Mid-electrode only
EfficientNet-B3 (weighted) *	0.30	90.6	69.2 [42.4–87.3]	92.5 [87.4–95.9]	45.0	97.1	0.545	0.919 [0.816–1.000]	9.2	0.33	Focal basal electrode only
DenseNet121 (weighted) *	0.30	90.6	53.8 [29.1–76.8]	93.8 [89.1–96.8]	43.8	95.8	0.483	0.908 [0.798–1.000]	8.7	0.49	Peripheral non-electrode
EfficientNet-B3 (non-weighted)	0.50	91.2	30.8 [12.7–57.6]	96.6 [92.5–98.6]	44.4	94.0	0.364	0.876 [0.752–1.000]	9.1	0.72	Apical electrode only

**Table 3:** Five-fold cross-validation results (mean ± standard deviation across folds) for all eight model configurations. Each test fold contained approximately 135 patients and 16–17 abnormal cases.

Model	Weight Strategy	Sensitivity	Specificity	PPV	NPV	F1-Score	AUC
ResNet18	non-weighted	27.9% ± 24.2%	94.6% ± 2.4%	25.6% ± 19.9%	94.2% ± 1.7%	0.263 ± 0.219	0.796 ± 0.076
ResNet18	weighted	57.7% ± 22.8%	87.6% ± 6.6%	29.2% ± 8.7%	96.2% ± 2.0%	0.374 ± 0.111	0.861 ± 0.030
EfficientNet-B0	non-weighted	29.0% ± 12.3%	95.1% ± 2.0%	33.2% ± 11.3%	94.2% ± 1.1%	0.301 ± 0.101	0.737 ± 0.093
EfficientNet-B0	weighted	49.8% ± 22.7%	88.5% ± 5.1%	27.9% ± 13.6%	95.5% ± 2.1%	0.349 ± 0.163	0.782 ± 0.110
DenseNet121	non-weighted	34.4% ± 15.1%	97.3% ± 1.1%	51.1% ± 16.2%	94.7% ± 1.4%	0.404 ± 0.142	0.835 ± 0.061
DenseNet121	weighted	58.4% ± 27.1%	92.7% ± 3.6%	42.3% ± 25.7%	96.4% ± 2.4%	0.475 ± 0.229	0.829 ± 0.129
EfficientNet-B3	non-weighted	45.4% ± 26.0%	93.5% ± 5.2%	37.7% ± 15.0%	95.4% ± 2.1%	0.382 ± 0.172	0.787 ± 0.100
EfficientNet-B3	weighted	50.5% ± 26.8%	93.3% ± 2.0%	36.6% ± 10.5%	95.8% ± 2.4%	0.414 ± 0.154	0.855 ± 0.117

**Table 4:** Complete pairwise AUC comparisons across all eight model configurations (28 comparisons), based on paired *t*-tests across the five cross-validation folds, with raw and Holm–Bonferroni-adjusted *p*-values. No comparison remained significant after correction for multiple testing.

Model A	Model B	Mean AUC A	Mean AUC B	Raw *p*	Holm-Adj. *p*
DenseNet121 (non-weighted)	DenseNet121 (weighted)	0.835	0.829	0.899	1.000
DenseNet121 (non-weighted)	EfficientNet-B3 (non-weighted)	0.835	0.787	0.340	1.000
DenseNet121 (non-weighted)	EfficientNet-B3 (weighted)	0.835	0.855	0.718	1.000
DenseNet121 (weighted)	EfficientNet-B3 (non-weighted)	0.829	0.787	0.288	1.000
DenseNet121 (weighted)	EfficientNet-B3 (weighted)	0.829	0.855	0.570	1.000
EfficientNet-B0 (non-weighted)	DenseNet121 (non-weighted)	0.737	0.835	0.046	1.000
EfficientNet-B0 (non-weighted)	DenseNet121 (weighted)	0.737	0.829	0.036	0.938
EfficientNet-B0 (non-weighted)	EfficientNet-B0 (weighted)	0.737	0.782	0.219	1.000
EfficientNet-B0 (non-weighted)	EfficientNet-B3 (non-weighted)	0.737	0.787	0.309	1.000
EfficientNet-B0 (non-weighted)	EfficientNet-B3 (weighted)	0.737	0.855	0.004	0.123
EfficientNet-B0 (weighted)	DenseNet121 (non-weighted)	0.782	0.835	0.324	1.000
EfficientNet-B0 (weighted)	DenseNet121 (weighted)	0.782	0.829	0.341	1.000
EfficientNet-B0 (weighted)	EfficientNet-B3 (non-weighted)	0.782	0.787	0.928	1.000
EfficientNet-B0 (weighted)	EfficientNet-B3 (weighted)	0.782	0.855	0.079	1.000
EfficientNet-B3 (non-weighted)	EfficientNet-B3 (weighted)	0.787	0.855	0.246	1.000
ResNet18 (non-weighted)	DenseNet121 (non-weighted)	0.796	0.835	0.080	1.000
ResNet18 (non-weighted)	DenseNet121 (weighted)	0.796	0.829	0.396	1.000
ResNet18 (non-weighted)	EfficientNet-B0 (non-weighted)	0.796	0.737	0.062	1.000
ResNet18 (non-weighted)	EfficientNet-B0 (weighted)	0.796	0.782	0.692	1.000
ResNet18 (non-weighted)	EfficientNet-B3 (non-weighted)	0.796	0.787	0.846	1.000
ResNet18 (non-weighted)	EfficientNet-B3 (weighted)	0.796	0.855	0.223	1.000
ResNet18 (non-weighted)	ResNet18 (weighted)	0.796	0.861	0.053	1.000
ResNet18 (weighted)	DenseNet121 (non-weighted)	0.861	0.835	0.214	1.000
ResNet18 (weighted)	DenseNet121 (weighted)	0.861	0.829	0.534	1.000
ResNet18 (weighted)	EfficientNet-B0 (non-weighted)	0.861	0.737	0.016	0.430
ResNet18 (weighted)	EfficientNet-B0 (weighted)	0.861	0.782	0.156	1.000
ResNet18 (weighted)	EfficientNet-B3 (non-weighted)	0.861	0.787	0.147	1.000
ResNet18 (weighted)	EfficientNet-B3 (weighted)	0.861	0.855	0.904	1.000

## Data Availability

The dataset used in this study contains identifiable patient information and cannot be made publicly available owing to institutional data governance regulations and IRB restrictions. De-identified data may be available upon reasonable request to the corresponding author, subject to approval from the KAESC Institutional Review Board. Code Availability Statement; The model training pipeline and evaluation scripts are not publicly available but may be shared upon reasonable request to the corresponding author. All models were implemented in PyTorch 2.13.0 and trained using Python 3.10 in Google Colab.

## Supplementary Materials

The following supporting information can be downloaded at: https://www.mdpi.com/article/10.3390/jcm15156108/s1, Supplementary File S1: CLAIM 2024 checklist for AI in medical imaging, mapped to the manuscript. Supplementary File S2: TRIPOD+AI checklist for prediction model reporting, mapped to the manuscript. References [31,32] are cited in the supplementary materials.
