# Supplementary material for: Artificial Intelligence Reading of Postoperative Cochlear Implant X-Rays
Source: J Clin Med. 2026 Aug 6;15(15):6108. doi: 10.3390/jcm15156108 (PMC13467076; doi:10.3390/jcm15156108)
Supplement: Supplementary file 1 [file jcm-15-06108-s001.zip › Supplementary_File_S1_CLAIM_2024.pdf]

Supplementary File S1

CLAIM 2024 — Checklist for Artificial Intelligence in Medical Imaging

Artificial Intelligence Reading of Postoperative Cochlear Implant X-Rays

The following checklist maps each item of the CLAIM 2024 Update (Tejani et al., Radiology: Artificial Intelligence, 2024) to its location in the manuscript. Items not applicable to a retrospective proof-of-concept classification study are marked N/A with a brief justification.

#	Checklist Item	Reported	Location in Manuscript
**TITLE AND ABSTRACT**			
1	Identification as a study of AI methodology, specifying the category of technology used (e.g., deep learning).	**Yes**	Title; Abstract (Background/Objectives)
2	Structured summary of study design, methods, results, and conclusions.	**Yes**	Abstract (structured: Background/Objectives, Methods, Results, Conclusions)
**INTRODUCTION**			
3	Scientific and clinical background, including the intended use and clinical role of the AI approach.	**Yes**	Introduction, paragraphs 1–2 (clinical role: postoperative electrode-position screening)
4	Study objectives and hypotheses.	**Yes**	Introduction, final paragraph
**METHODS — Study Design**			
5	Prospective or retrospective study.	**Yes**	Section 2.1 (retrospective)
6	Study goal, such as model creation, exploratory study, feasibility study, non-inferiority trial.	**Yes**	Abstract; Title (proof-of-concept / feasibility); Section 2.1
**METHODS — Data**			
7	Data sources.	**Yes**	Section 2.1–2.2 (single tertiary referral center, KAESC)
8	Eligibility criteria: how, where, and when potentially eligible participants or studies were identified.	**Yes**	Section 2.2 (inclusion/exclusion criteria; study period)
9	Data pre-processing steps.	**Yes**	Section 2.4 (ROI annotation, cropping, resizing to 256×256, intensity normalization)
10	Selection of data subsets, if applicable.	**Yes**	Section 2.5 (patient-level 70/15/15 split); Section 3.1 (cohort flow)
11	Definitions of data elements, with references to Common Data Elements.	**Yes**	Section 2.3 (label definitions: normal vs abnormal, with four-category origin)
12	De-identification methods.	**Yes**	Institutional Review Board / Informed Consent statements (anonymized imaging data)
13	How missing data were handled.	**Yes**	Section 3.1 (two cases with incomplete labels excluded)
**METHODS — Ground Truth (Reference Standard)**			
14	Definition of ground truth reference standard, in sufficient detail to allow replication.	**Yes**	Section 2.3 (masked review by two senior specialists; consensus)
15	Rationale for choosing the reference standard.	**Yes**	Section 2.3 (expert consensus as clinical reference standard)
16	Source of ground-truth annotations; qualifications and preparation of annotators.	**Yes**	Section 2.3 (two independent senior specialists)
17	Annotation tools.	**Yes**	Section 2.4 (LabelImg)
18	Measurement of inter- and intra-rater variability; methods to mitigate variability and/or resolve discrepancies.	Partial	Section 2.3 (discrepancies resolved by consensus; inter-rater statistic not computed — see Limitations)

#	Checklist Item	Reported	Location in Manuscript
**METHODS — Data Partitions**			
19	Intended sample size and how it was determined.	**Yes**	Section 4 / Limitations (Buderer-based sample-size discussion)
20	How data were assigned to partitions; specify proportions.	**Yes**	Section 2.5 (patient-level stratified 70/15/15; bilateral cases kept together)
21	Level at which partitions are disjoint (e.g., image, study, patient).	**Yes**	Section 2.5 (patient level — no leakage across folds)
**METHODS — Model**			
22	Detailed description of model, including inputs, outputs, and all intermediate layers and connections.	**Yes**	Section 2.5 (ResNet18, EfficientNet-B0, DenseNet121, EfficientNet-B3 via transfer learning)
23	Software libraries, frameworks, and packages.	**Yes**	Section 2.5 (PyTorch; Python; Google Colab)
24	Initialization of model parameters.	**Yes**	Section 2.5 (ImageNet-pretrained transfer learning)
**METHODS — Training**			
25	Details of training approach, including data augmentation, hyperparameters, number of models trained.	**Yes**	Section 2.5 (AdamW; LR 1×10^{-4}; batch 16; 20 epochs; weighted vs non-weighted; 8 configurations)
26	Method of selecting the final model.	**Yes**	Section 2.5–2.6 (validation F1-based threshold tuning; cross-validation for generalizability)
27	Ensembling techniques, if applicable.	N/A	No ensembling used; architectures compared individually
**METHODS — Evaluation**			
28	Metrics of model performance.	**Yes**	Section 2.6; Section 3.1 (accuracy, sensitivity, specificity, PPV, NPV, F1, AUC, LR+, LR−)
29	Statistical measures of significance and uncertainty (e.g., confidence intervals).	**Yes**	Section 3.1–3.3 (95% CIs; paired t-tests across folds)
30	Robustness or sensitivity analysis.	**Yes**	Section 3.2 (five-fold cross-validation); Section 3.3 (statistical comparisons)
31	Methods for explainability or interpretability (e.g., saliency maps).	**Yes**	Section 2.6; Section 3.1 (Grad-CAM); Figure 4
32	Validation or testing on external data.	No	Single-center study; external validation identified as required next step (Section 4, Conclusions)
**RESULTS**			
33	Flow of participants or cases, using a diagram to indicate inclusion and exclusion.	**Yes**	Figure 3 (STARD flow diagram); Section 3.1
34	Demographic and clinical characteristics of cases in each partition.	Partial	Section 3.1 (cohort counts and prevalence; detailed demographics limited by retrospective scope)
35	Performance metrics for optimal model(s) on all data partitions.	**Yes**	Section 3.1–3.2; Table 1; Table 2
36	Estimates of diagnostic accuracy and their precision (such as 95% confidence intervals).	**Yes**	Table 1 (95% CIs, LR+, LR−); Section 3.1
37	Failure analysis of incorrectly classified cases.	Partial	Section 3.1 / Discussion (Grad-CAM identifies spurious-feature reliance in EfficientNet models)
**DISCUSSION**			
38	Study limitations, including potential bias, statistical uncertainty, and generalizability.	**Yes**	Section 4 (Limitations: class imbalance, threshold transfer, single-center, annotation)

#	Checklist Item	Reported	Location in Manuscript
39	Implications for practice, including the intended use and/or clinical role.	**Yes**	Section 4 (triage/screening-support role; LR interpretation); Conclusions
**OTHER INFORMATION**			
40	Registration number and name of registry.	N/A	Not a clinical trial; retrospective diagnostic study
41	Where the full study protocol can be accessed.	N/A	No separate protocol; methods fully described in Section 2
42	Sources of funding and other support; role of funders.	**Yes**	Funding Statement (no external funding)

Reference: *Tejani AS, Klontzas ME, Gatti AA, et al. Checklist for Artificial Intelligence in Medical Imaging (CLAIM): 2024 Update. Radiol Artif Intell. 2024;6(4):e240300.*
