# Supplementary material for: Artificial Intelligence Reading of Postoperative Cochlear Implant X-Rays
Source: J Clin Med. 2026 Aug 6;15(15):6108. doi: 10.3390/jcm15156108 (PMC13467076; doi:10.3390/jcm15156108)
Supplement: Supplementary file 1 [file jcm-15-06108-s001.zip › Supplementary_File_S2_TRIPOD_AI.pdf]

Supplementary File S2

TRIPOD+AI — Transparent Reporting of a Multivariable Prediction Model for Individual Prognosis or Diagnosis (AI Extension)

Artificial Intelligence Reading of Postoperative Cochlear Implant X-Rays

This checklist maps each TRIPOD+AI item (Collins et al., BMJ, 2024) to its location in the manuscript. As a model-development study without external validation, development-specific items are completed; external-validation items are marked accordingly.

#	TRIPOD+AI Checklist Item	Reported	Location in Manuscript
**TITLE AND ABSTRACT**			
1	Identify the study as developing and/or evaluating a multivariable prediction model, the target population, and the outcome to be predicted.	**Yes**	Title; Abstract
2	Provide a structured summary of objectives, study design, setting, participants, sample size, predictors, outcome, statistical analysis, results, and conclusions.	**Yes**	Abstract (structured)
**INTRODUCTION — Background and Objectives**			
3	Explain the medical context and rationale for developing or evaluating the model, including references to existing models.	**Yes**	Introduction, paragraphs 1–3
4	Describe the objectives, including whether the study describes model development, validation, or both.	**Yes**	Introduction, final paragraph (development/evaluation, proof-of-concept)
**METHODS — Source of Data**			
5	Describe the study design and key dates (start of accrual; end of follow-up).	**Yes**	Section 2.1–2.2 (retrospective; study period)
6	Specify key elements of the study setting (e.g., number and location of centers).	**Yes**	Section 2.1 (single tertiary referral center, KAESC, Riyadh)
**METHODS — Participants**			
7	Describe eligibility criteria for participants.	**Yes**	Section 2.2
8	Report details of any treatments received, if relevant.	N/A	Diagnostic imaging classification; no treatment exposure relevant
**METHODS — Outcome**			
9	Clearly define the outcome that is predicted, including how and when assessed.	**Yes**	Section 2.3 (binary: normal vs abnormal electrode position; expert consensus)
10	Report any actions to blind assessment of the outcome to be predicted.	**Yes**	Section 2.3 (masked specialist review)
**METHODS — Predictors**			
11	Describe predictors used, including how and when measured.	**Yes**	Section 2.4 (radiographic ROI image features learned by CNNs)
12	Report any actions to blind assessment of predictors for the outcome and other predictors.	**Yes**	Section 2.4–2.5 (automated feature extraction; no outcome leakage)
**METHODS — Sample Size and Missing Data**			
13	Explain how the study size was arrived at.	**Yes**	Section 4 / Limitations (Buderer-based discussion)
14	Describe how missing data were handled.	**Yes**	Section 3.1 (two incomplete-label cases excluded)
**METHODS — Analytical Methods**			

#	TRIPOD+AI Checklist Item	Reported	Location in Manuscript
15	Describe how predictors were handled in the analyses.	**Yes**	Section 2.4–2.5 (image preprocessing; transfer-learning feature extraction)
16	Specify the type of model, all model-building procedures, and method for internal validation.	**Yes**	Section 2.5–2.6 (four CNNs; weighting; threshold tuning; five-fold CV)
17	Specify how AI-specific elements (architecture, training, hyperparameters, computational resources) were addressed.	**Yes**	Section 2.5 (architectures; AdamW; LR; epochs; PyTorch; Colab)
18	Describe measures used to assess model performance and, if relevant, to compare multiple models.	**Yes**	Section 2.6; Section 3.3 (metrics; paired t-tests across folds)
19	Describe any model updating arising from validation, if applicable.	N/A	No external validation/updating in this proof-of-concept
**METHODS — Fairness, Ethics, and Transparency**			
20	Report how the model accounts for, or was assessed for, fairness across subgroups.	Partial	Class-imbalance handling reported (Section 2.5); subgroup fairness not formally assessed (Limitations)
21	Provide details of ethical approval and participant consent.	**Yes**	Institutional Review Board Statement; Informed Consent Statement
**RESULTS — Participants**			
22	Describe the flow of participants, including the number with and without the outcome.	**Yes**	Figure 3 (STARD); Section 3.1 (949 normal, 84 abnormal)
23	Report characteristics of participants and the number in each analysis.	Partial	Section 3.1 (cohort and partition counts; prevalence 8.1%)
**RESULTS — Model Development and Performance**			
24	Specify the number of participants and outcome events in each analysis.	**Yes**	Section 3.1–3.2 (per-partition and per-fold counts)
25	Report model performance estimates with confidence intervals; for AI, report performance across operating points if relevant.	**Yes**	Table 1 (95% CIs, thresholds, LR+/LR−); Table 2 (CV mean ± SD)
26	If done, report results of any comparison of multiple models or with existing models.	**Yes**	Section 3.3 (pairwise AUC comparisons; weighted vs non-weighted)
27	Provide details for model interpretability/explainability.	**Yes**	Section 3.1 (Grad-CAM); Figure 4
**DISCUSSION**			
28	Give an overall interpretation of the results in the context of objectives and prior evidence.	**Yes**	Section 4, paragraphs 1–3
29	Discuss limitations (e.g., non-representative sample, few events, overfitting, missing data).	**Yes**	Section 4 (Limitations)
30	Discuss generalizability, clinical usefulness, and intended use of the model.	**Yes**	Section 4 (LR-based clinical interpretation; triage role); Conclusions
**OTHER INFORMATION**			
31	Provide information about supplementary resources (code, data, model).	**Yes**	Data Availability and Code Availability Statements
32	Give the source of funding and the role of funders.	**Yes**	Funding Statement (none)
33	Report the availability of the study protocol and registration.	N/A	No separate protocol or registration (retrospective diagnostic study)

Reference: *Collins GS, Moons KGM, Dhiman P, et al. TRIPOD+AI statement: updated guidance for reporting clinical prediction models that use regression or machine learning methods. BMJ. 2024;385:e078378.*
